# Influence of native ureolytic microbial community on biocementation potential of *Sporosarcina pasteurii*

**DOI:** 10.1038/s41598-021-00315-5

**Published:** 2021-10-21

**Authors:** Raja Murugan, G. K. Suraishkumar, Abhijit Mukherjee, Navdeep K. Dhami

**Affiliations:** 1 Bhupat and Jyoti Mehta School of Biosciences, Indian Institute of Technology Madras, Chennai, 600036 India; 2 School of Civil and Mechanical Engineering, Curtin University, Perth, WA 6102 Australia

**Keywords:** Biotechnology, Biogeochemistry

## Abstract

Microbially induced calcium carbonate precipitation (MICP)/Biocementation has emerged as a promising technique for soil engineering applications. There are chiefly two methods by which MICP is applied for field applications including biostimulation and bioaugmentation. Although bioaugmentation strategy using efficient ureolytic biocementing culture of *Sporosarcina pasteurii* is widely practiced, the impact of native ureolytic microbial communities (NUMC) on CaCO_3_ mineralisation via *S. pasteurii* has not been explored. In this paper, we investigated the effect of different concentrations of NUMC on MICP kinetics and biomineral properties in the presence and absence of *S. pasteurii.* Kinetic analysis showed that the biocementation potential of *S. pasteurii* is sixfold higher than NUMC and is not significantly impacted even when the concentration of the NUMC is eight times higher. Micrographic results revealed a quick rate of CaCO_3_ precipitation by *S. pasteurii* leading to generation of smaller CaCO_3_ crystals (5–40 µm), while slow rate of CaCO_3_ precipitation by NUMC led to creation of larger CaCO_3_ crystals (35–100 µm). Mineralogical results showed the predominance of calcite phase in both sets. The outcome of current study is crucial for tailor-made applications of MICP.

## Introduction

Microbially induced calcium carbonate precipitation (MICP) is a ubiquitously recorded process in nature and is responsible for creation of numerous geological formations in terrestrial and marine environments^1^. Recently this process has been replicated in lab conditions for numerous engineering applications, as it leads to the formation of carbonate cement at ambient temperature conditions by harnessing the cementation potential of living microorganisms. The major applications include improvement of mechanical properties of soil^2,3^, bioremediation of heavy metals and radio nucleotides^4–6^, enhancement of oil recovery^7^, repair of concrete cracks^8,9^, and sequestration of atmospheric CO_2_^10^_._ The chief benefit of this bio-mimicked cementation process includes self-healing ability, eco-friendliness, recyclability, and low viscosity paving the way for deeper penetration^11^.

MICP/Biocementation occurs via various metabolic pathways of bacteria such as ureolysis, denitrification, sulfate reduction, and iron reduction^12^. Amongst the different pathways, MICP via ureolytic pathway is the most widely explored route because of its straightforwardness, efficacy, short time, and no excess production of protons^13,14^. In the microbial ureolytic pathway, urea is hydrolysed into ammonia and carbon dioxide by the action of urease^2^. Subsequently, these products equilibrate in water to form bicarbonate, ammonium, and hydroxide ions, which elevate the pH of the microenvironment around the bacteria (Eq. 1). An increase in pH favors the equilibrium shifts from bicarbonate ions to carbonate ions. The formed carbonate ions then precipitate as calcium carbonate on the bacterial surface in the presence of calcium^2^ (Eq. 2).

1 $$Co(N{H}_{2}{)}_{2} + 2 {H}_{2}O \to HC{O}_{3}^{2-} + 2N{H}_{4 }^{+}+ {OH}^{-}$$

2 $${\mathrm{C}{a}^{2+} +\mathrm{ C}O}_{3}^{2- }+bacteria \to bacteria- CaC{O}_{3}\downarrow $$

For applications of MICP in soils, especially in the field, there are two modes by which calcifying bacteria are supplemented: biostimulation (enrichment of native population) or bioaugmentation (supplementation of efficient foreign bacteria). The biostimulation approach deals with the modification of existing field conditions by altering the nutrients, substrates, and electron acceptors to enrich the native microorganisms for accelerating the CaCO_3_ precipitation; whereas, bioaugmentation includes the addition of highly potential ureolytic and cementing strains especially *Sporosarcina pasteurii* into the fields^5,32–35^. Comparing these two approaches, MICP through bioaugmentation has a major advantage as it is a rapid process. This benefit makes this approach quite attractive for engineering applications, despite having the limitation of cost factor for preparation and transport of bacterial cultures^34^. On the other hand, biostimulation utilizes native bacteria making the MICP process both economically and environmentally viable^34^. Furthermore, the stimulation approach may eliminate the possible ecological impacts caused by a non-indigenous bacterial introduction in the applied soil environment, but the process rate is generally slow in comparison to the bioaugmentation approach^36^. The studies conducted on utilisation of both the approaches for improving the soil engineering properties reported that changes in solution chemistry and distribution of CaCO_3_ precipitate occurred invariably in 1-m soil column during biostimulation^36^; however, bioaugmentation with *S. pasteurii* led to significant improvement in strength, stiffness, load-bearing capacity and hydraulic conductivity of the soil^12,36,37^. Although researchers have demonstrated biogeochemical changes during the biostimulation approach, not much has been investigated on the impact of native ureolytic microbial communities (NUMC) on the performance of *S. pasteurii* and how these communities perform in comparison to this high urease producing culture^36^. Also, the concentration of NUMC changes vastly in the field and may affect the kinetics of the CaCO_3_ process, its mineralogy, and morphology which are the determining factors for the success of biocementation^22^.

Theoretical quantification methods and kinetic models are useful to predict the efficacy of the MICP process and to develop practical guidelines for field applications. There are studies in which theoretical models have been developed to quantify the CaCO_3_ precipitation efficacy in soil engineering^56^ and crack repair applications^58^. In MICP, the kinetics of the CaCO_3_ precipitation depends on the urease production of the bacteria and decides the overall efficacy of biocementation. Both kinetics of CaCO_3_ precipitation and urease production of the bacteria are influenced by both abiotic and biotic factors including temperature, pH, aeration, nutrient availability, bacterial concentration, and type of bacteria or microbial population^15–21^. For instance, the urease activity and rate of CaCO_3_ precipitation at 30 ºC were reported to be higher than the observations at 15 ºC^54^. Besides, aerated conditions yielded a higher percentage of CaCO_3_ than unaerated conditions^20^_,_ and the study also attempted to increase the urease production for improving the process by changing the nutrient concentration, especially urea (0 to 40 g/L) in the medium^55^. The influence of concentration of bacteria and urease enzyme is reflected in the kinetic constant of the CaCO_3_ precipitation in terms of first-order rate constant of 0.002 to 0.60 h^-1^^22–25^. Further, the kinetics of the process also control the morphological and nanomechanical properties of the precipitated CaCO_3_; the slow rate of precipitation leads to the production of larger-sized crystals that are relatively stable compared to the smaller crystals formed at a high rate of precipitation^26,27^.

In general, microbially induced CaCO_3_ precipitate is a cohesive material^2^ and exists in different crystalline phases including calcite, vaterite, aragonite, monohydrocalcite, and ikaite^1^. The sizes of these crystals vary from 5 to 100 µm along with variations in their nanomaterial properties^28–30^. Essential properties such as size, shape, stability, solubility, and hardness of the CaCO_3_ crystals determine the efficacy of MICP in engineering applications. For example, the conservation of building materials required more stable calcite than metastable vaterite and larger rhombohedral crystals (100–150 µm) are more preferable in soil stabilization applications^31^. Even though studies utilized biostimulation and/or bioaugmentation approaches in MICP applications^32–38^, but very little information is available on these aspects including the impact of native ureolytic communities on MICP kinetics with and without *S. pasteurii*, the effect of the concentration of native communities on MICP, and the influence of kinetic factors on morpho-mineralogical properties of carbonate crystals. All these factors are crucial in determining the efficacy of biocementation for field applications. The purpose of this study is to (1) evaluate the influence of native ureolytic microbial community (NUMC) at varying concentrations on biocementation kinetics, (2) analyse the bioaugmentation potential of *S. pasteurii* in presence of different concentrations of native ureolytic microbial community and (3) investigate the effect of different cell concentrations of NUMC on morphological-mineralogical properties of *S. pasteurii* driven MICP.

We hypothesize that the outcome of this study will help to tailor MICP kinetics, morphology, mineralogy, and material properties of biomineralised crystals via both the stimulation and augmentation approach.

## Results

### Influence of the native ureolytic microbial community on the kinetics of calcium carbonate precipitation

**Figure 1:**
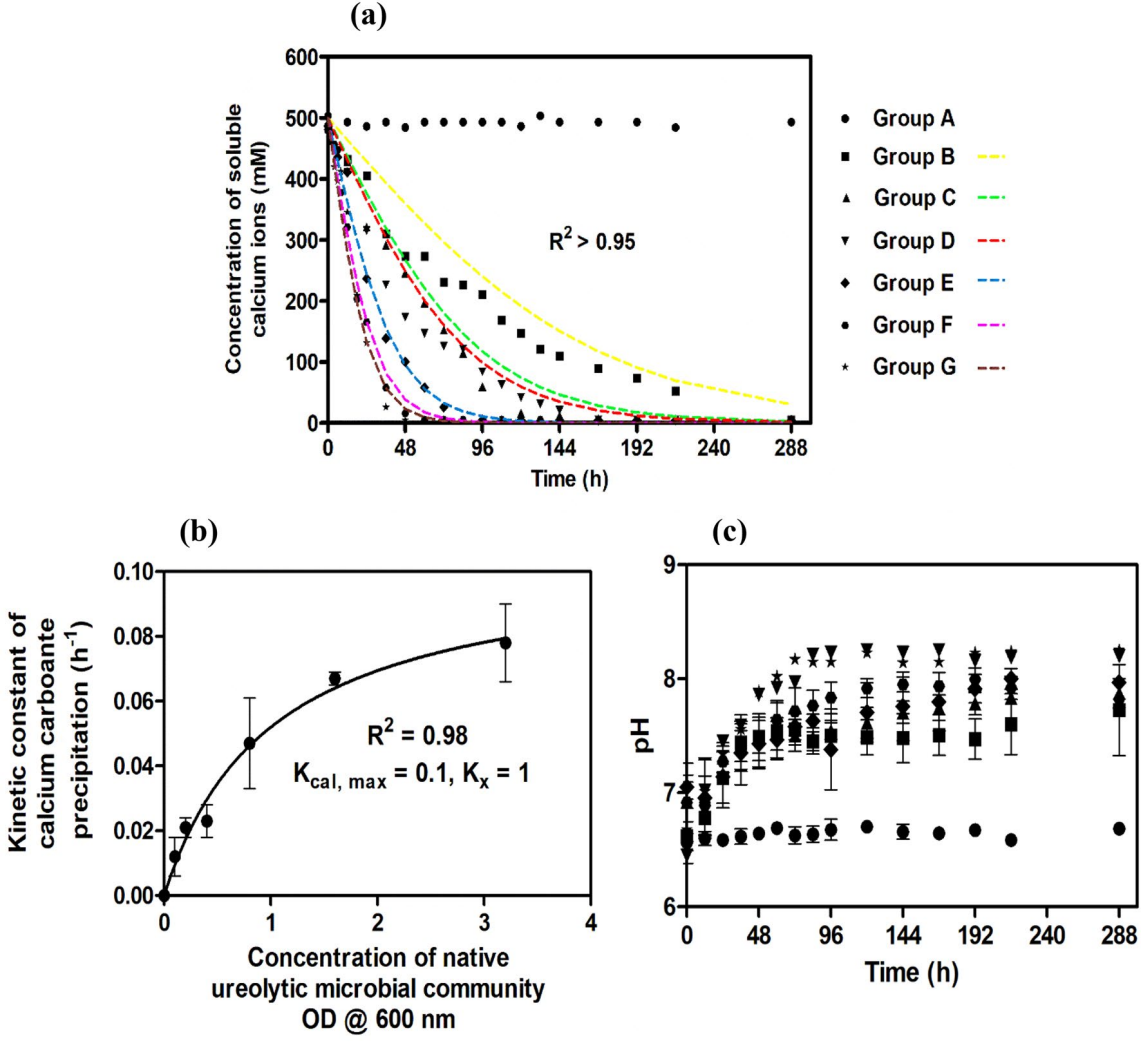
Concentration of soluble calcium ions over time **(a)**. The relationship between the kinetic constant of CaCO_3_ precipitation and concentration of the native ureolytic microbial community **(b)**. The variation of pH with time **(c)**. Group A—0 OD NUMC, Group B—0.1 OD NUMC, Group C—0.2 OD NUMC, Group D—0.4 OD NUMC, Group E—0.8 OD NUMC, Group F—1.6 OD NUMC, and Group G—3.2 OD NUMC. *NUMC* Native Ureolytic Microbial Community. The coloured hidden lines are computationally fitted curves. Error bars in the figure indicate the standard deviation of three independent trials.

To investigate the influence of native ureolytic microbial community (NUMC) on calcium carbonate precipitation at varying concentrations (0, 0.1, 0.2, 0.4, 0.8, 1.6, and 3.2 OD), soluble calcium concentration in the cementation medium was monitored for up to 288 h (at an interval of 12 h). From Fig. 1a it can be observed that the soluble calcium concentration decreased over time in all the groups with varying rates except group A to which no NUMC was added. The calcium concentration decreased to 50% from the initial value for group B at 96th hour, for group C at 60th hour, for group D at 48th hour, for group E at 36th hour, and group F and G at the 24th hour. At the end of the process, the soluble calcium ions in all the sets were exhausted, except in set A.

Kinetic constants (K_cal_) of CaCO_3_ precipitation were used to further investigate the effect of various parameters on carbonate precipitation^35^. The monitored profiles were computationally fitted using Eq. (4) to calculate K_cal_ values (Fig. 1a). Table 1 shows K_cal_ values at varying NUMC. It can be seen that the K_cal_ values increased from group A (0 h^−1^) to group G (0.078 h^−1^) with increasing initial NUMC concentration. Further, the K_cal_ values can be described by Michelis-Menten (MM) type Eq. (3).

3 $${K}_{cal}=\frac{{K}_{cal, max} X}{{K}_{x}+X} $$

where K_x_ is a constant, X is the NUMC concentration, and K_cal, max_ is the maximum kinetic constant for CaCO_3_ precipitation. When K_x_ is equal to X, the value of K_cal_ is equal to half of the K_cal, max_. The observed values are K_x_ = 1 OD and K_cal_, _max_ = 0.1 h^−1^ in this study. Figure 1b shows MM type plot that relates K_cal_ with NUMC concentration.

**Table 1:** The kinetic constant values of CaCO_3_ precipitation at varying native ureolytic microbial community concentration.

S. No	Native ureolytic microbial community Group ID	Concentration of NUMC OD @ 600 nm	Kinetic constant of calcium carbonate precipitation (h^−1^)	R^2^
1	Group A	0	0	NA
2	Group B	0.1	0.012 ± 0.006	0.97
3	Group C	0.2	0.021 ± 0.003	0.98
4	Group D	0.4	0.023 ± 0.005	0.98
5	Group E	0.8	0.047 ± 0.014	0.99
6	Group F	1.6	0.067 ± 0.002	0.99
7	Group G	3.2	0.078 ± 0.012	0.98

*NUMC* Native Ureolytic Microbial Community, *NA* not applicable.

± indicates the standard deviation of two independent trials.

Figure 1c shows the pH change over time in all the sets. In the cementation medium, pH was observed to be between 6.5 and 8.3 in all the groups throughout the process. It can be seen that the rate of pH change within the groups followed a similar trend except for the control group A.

### Influence of the native ureolytic microbial community on augmented *S. pasteurii*

**Figure 2:**
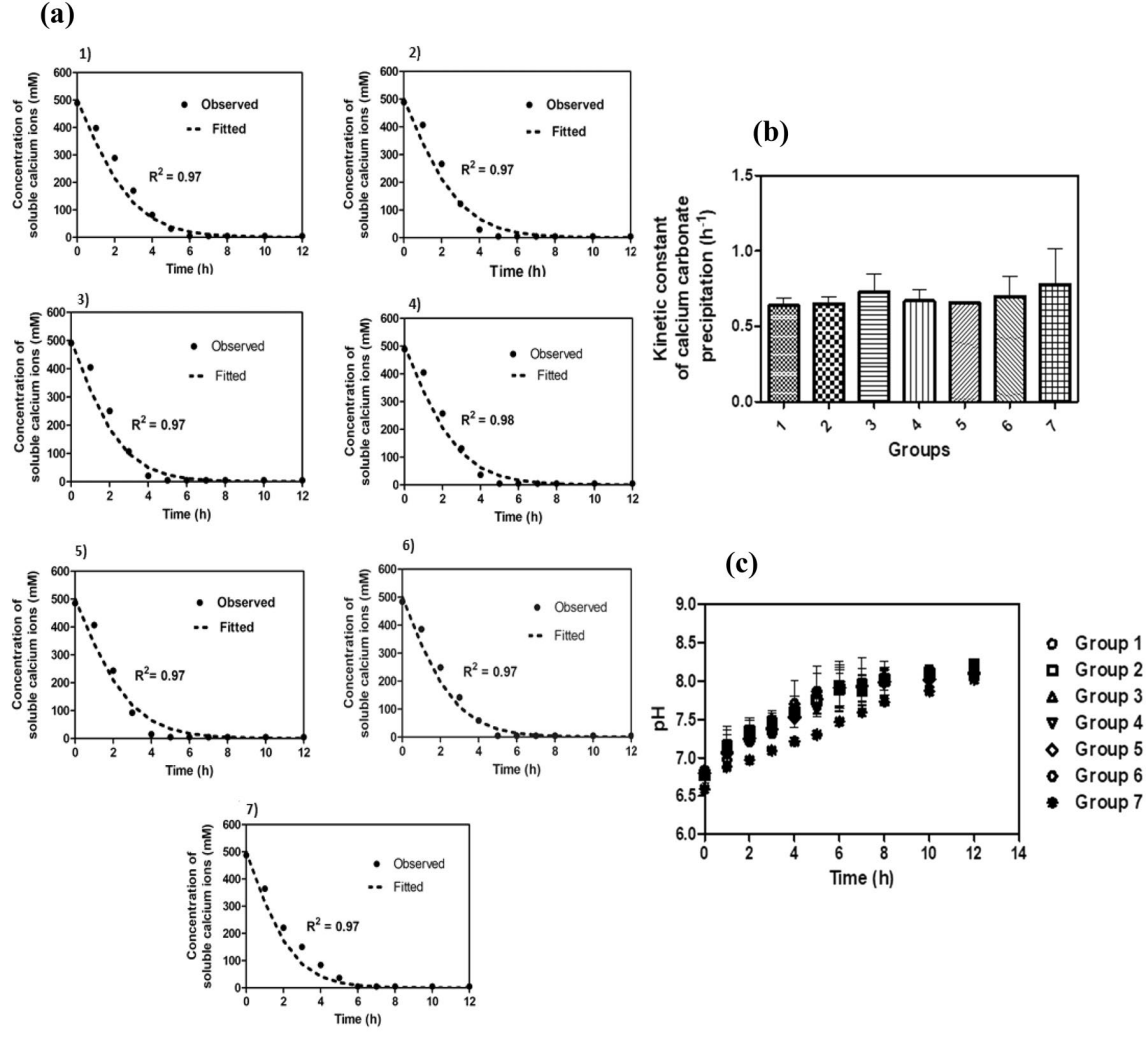
Concentration of soluble calcium ions over time—bioaugmentation** (a), **comparison of the kinetic constant of CaCO_3_ precipitation—bioaugmentation **(b)**, the variation of pH with time** (c)**. Group 1—0 OD NUMC + 0.4 OD *S*. *pasteurii*, Group 2—0.1 OD NUMC + 0.4 OD *S*. *pasteurii*, Group 3—0.2 OD NUMC + 0.4 OD *S*. *pasteurii*, 4) Group 4—0.4 OD NUMC + 0.4 OD *S*. *pasteurii*, 0.8 OD NUMC + 0.4 OD *S*. *pasteurii*, 1.6 OD NUMC + 0.4 OD *S*. *pasteurii*, and 3.2 OD NUMC + 0.4 OD *S*. *pasteurii*. *NUMC* Native Ureolytic Microbial Community. Bioaugmentation—(NUMC + *S. pasteurii*). Error bars in the Fig. 2b and 2c indicate the standard deviation of two independent trials.

To investigate the influence of NUMC on *S*. *pasteurii* (bioaugmentation), soluble calcium concentration in the cementation medium was monitored over time and fitted with Eq. (4). Figure 2a shows both observed and fitted curves from groups 1 to 7. From this figure, an exponential decrease of soluble calcium concentration was observed in all the groups with immediate effect upon the addition of NUMC and *S. pasteurii.* The concentration was recorded to be around zero at the 6th hour. From the fitted curves, the values of the kinetic constants for calcium carbonate precipitation were calculated (Table 2) and compared (Fig. 2b). From Table 2 it can be seen that the kinetic constant values are 0.64, 0.65, 0.64, 0.73, 0.67, 0.66, 0.70, and 0.78 h^−1^ for the groups 1 to 7, respectively, i.e., the values were distributed between 0.64 and 0.78 h^−1^. The change in the pH values of the cementation medium was also monitored (Fig. 2c) and the observed values were found to be between 6.5 and 8 for all the groups.

**Table 2:** The kinetic constants of calcium carbonate precipitation.

S. No	Group ID	Concentration of NUMC OD @ 600 nm	Concentration of *S*. *pasteurii* OD@ 600 nm	Kinetic constant of CaCO_3_ precipitation (h^−1^)	R^2^
1	Group 1	0	0.4	0.64 ± 0.05	0.97
2	Group 2	0.1		0.65 ± 0.05	0.97
3	Group 3	0.2		0.73 ± 0.12	0.97
4	Group 4	0.4		0.67 ± 0.07	0.98
5	Group 5	0.8		0.66 ± 0.00	0.97
6	Group 6	1.6		0.70 ± 0.13	0.97
7	Group 7	3.2		0.78 ± 0.24	0.97

± indicates the standard deviation of two independent trials.

*NUMC* Native Ureolytic Microbial Community, *S*. *pasteurii* *Sporosarcina pasteurii.*

### Morphology and Phase of CaCO_3_

**Figure 3:**
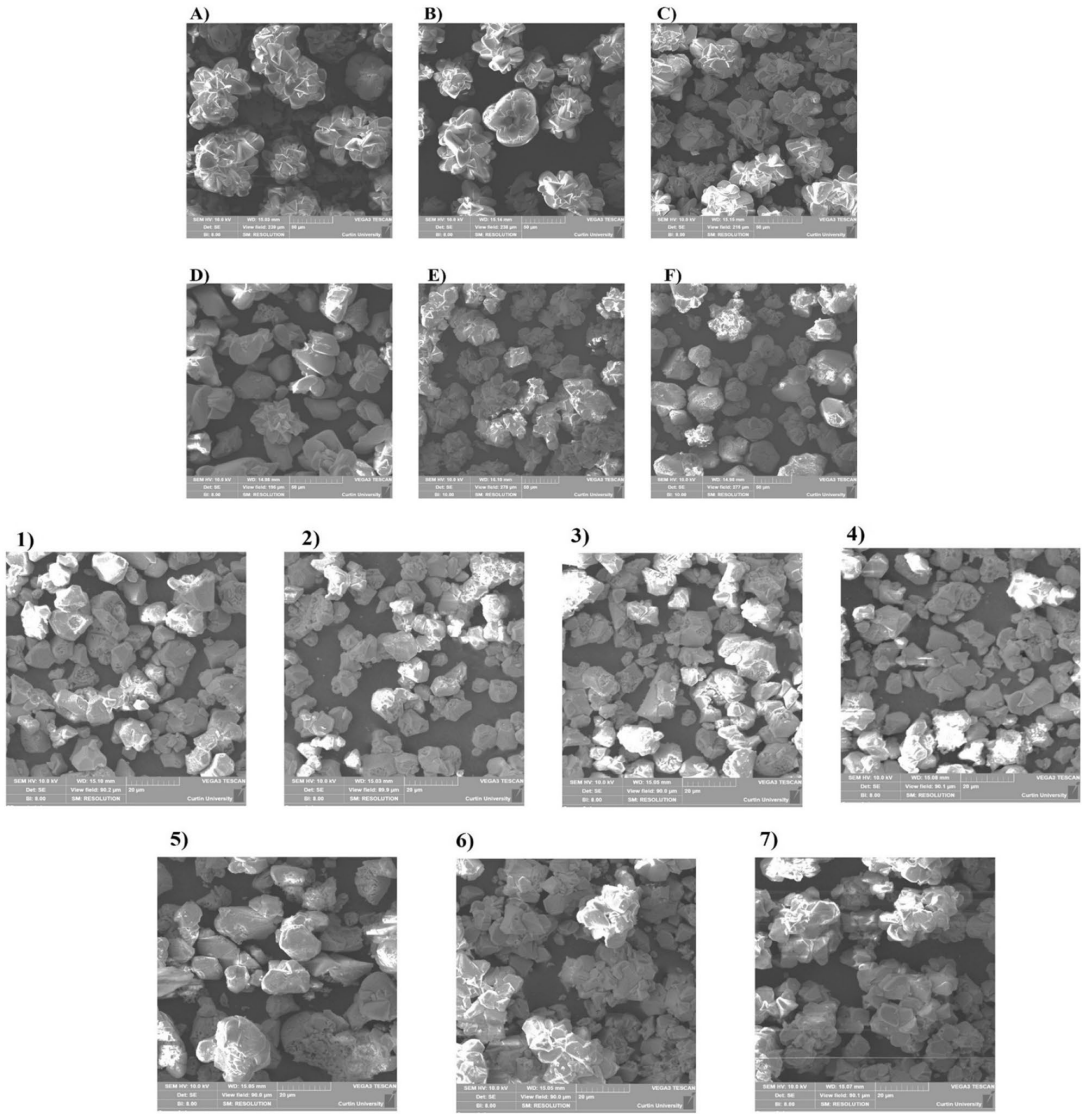
Scanning electron microscopy images of CaCO_3_ crystals. Groups B to G (NUMC) and 1 to 7 (bioaugmentation). (B)—0.1 OD NUMC, (C)—0.2 OD NUMC, (D)—0.4 OD NUMC, (E)—0.8 OD NUMC, (F)—1.6 OD NUMC, and (G)—3.2 OD. (1)—0 OD NUMC + 0.4 OD *S*. *pasteurii*, (2)—0.1 OD NUMC + 0.4 OD *S*. *pasteurii*, (3)—0.2 OD NUMC + 0.4 OD *S*. *pasteurii*, (4) Group 4—0.4 OD NUMC + 0.4 OD *S*. *pasteurii*, (5) 0.8 OD NUMC + 0.4 OD *S*. *pasteurii*, (6) 1.6 OD NUMC + 0.4 OD *S*. *pasteurii*, and (7) 3.2 OD NUMC + 0.4 OD *S*. *pasteurii*. *NUMC* Native Ureolytic Microbial Community and bioaugmentation—(NUMC + *S. pasteurii*).

**Figure 4:**
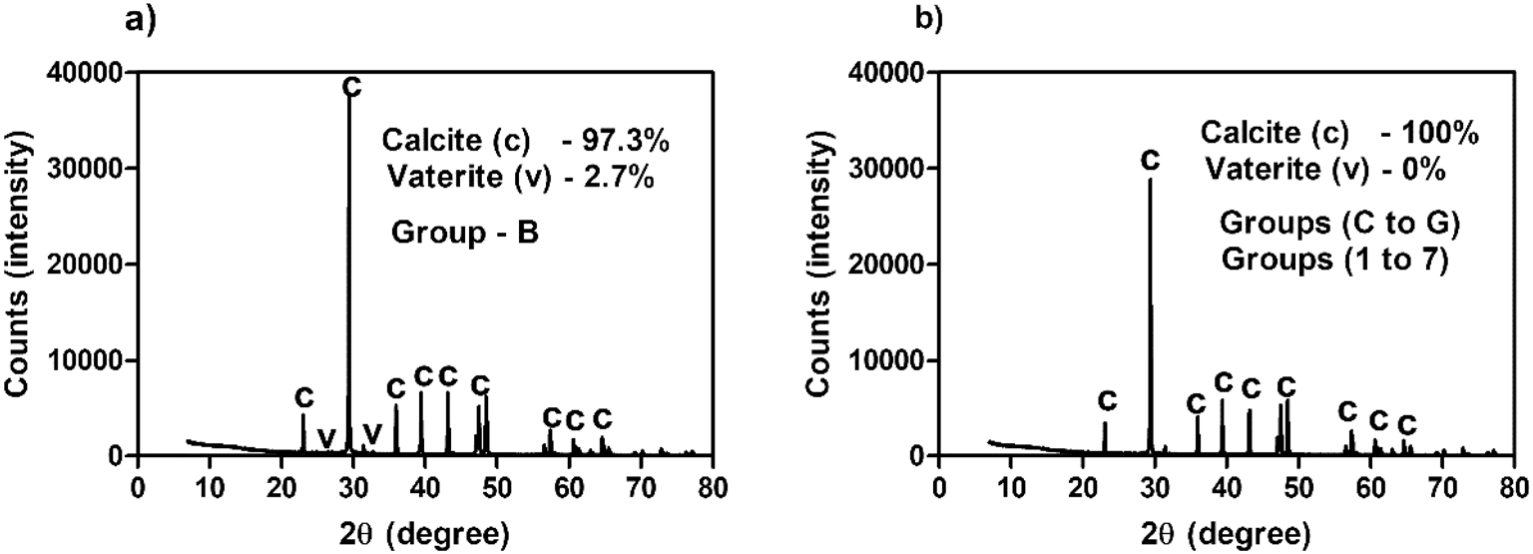
XRD spectrum of CaCO_3_ polymorphs. **(a)** Group B (NUMC) and **(b)** Groups C to G (NUMC) and 1 to 7 (bioaugmentation). Group B—0.1 OD NUMC, Group C—0.2 OD NUMC, Group D—0.4 OD NUMC, Group E—0.8 OD NUMC, Group F—1.6 OD NUMC, and Group G—3.2 OD NUMC. Group 1—0 OD NUMC + 0.4 OD *S*. *pasteurii*, Group 2—0.1 OD NUMC + 0.4 OD *S*. *pasteurii*, Group 3—0.2 OD NUMC + 0.4 OD *S*. *pasteurii*, (4) Group 4—0.4 OD NUMC + 0.4 OD *S*. *pasteurii*, 0.8 OD NUMC + 0.4 OD *S*. *pasteurii*, 1.6 OD NUMC + 0.4 OD *S*. *pasteurii*, and 3.2 OD NUMC + 0.4 OD *S*. *pasteurii*. *NUMC* Native Ureolytic Microbial Community, bioaugmentation—(NUMC + *S. pasteurii*).

CaCO_3_ crystal morphology varies depending on the surface properties of the bacterial cell wall composition especially extracellular polymeric substances and the solution chemistry of the medium^26^. Hence, the shape and size of precipitated crystals were analysed via scanning electron micrography (Fig. 3). For groups 1 to 4, rhombohedral-shaped crystals of size 5–10 µm were observed for the samples collected at the 12th hour. For group 5, the size of the individual and clustered rhombohedral-shaped crystals was found to be 15–25 µm for the samples collected at the 12th hour. For groups 6 and 7, for the samples collected at the 12th hour the size of both the clustered rhombohedral-shaped crystals was 30–40 µm. SEM images showed a cluster of rhombohedral-shaped crystals for the samples collected at 288th hour for the groups B to G. The size of these crystals varied between 35 and 100 µm. The polymorph is a determining factor of strength and hardness of CaCO_3_ in MICP. Therefore, the qualitative and quantitative information of the CaCO_3_ crystals were obtained using the powdered XRD technique (for the groups B to G at 288th hour and the groups 1 to 7 at the 12th hour). Figure 4 shows the XRD spectrum of group B and the representative spectrums of all the other groups. Tables 3 and 4 show the morphology and phase analysis of native ureolytic microbial community and bioaugmentation studies. It was observed that only group B showed 2.3% of the vaterite phase of CaCO_3_ crystals and all observed crystal phases of all the groups were of the calcite phase.

**Table 3:** The influence of native ureolytic microbial community concentration on the morphology and phase of CaCO_3_ crystals.

S. No	Group ID	Concentration of NUMC OD @ 600 nm	The average size of the crystal (µm)	Shape	Phase	
					Vaterite (%)	Calcite (%)
1	Group A	0	NA	NA	NA	NA
2	Group B	0.1	80–100	Cluster of rhombohedral	2.3	97.7
3	Group C	0.2	70–90	Cluster of rhombohedral	0	100
4	Group D	0.4	60–80	Cluster of rhombohedral	0	100
5	Group E	0.8	55–65	Individual and Cluster of rhombohedral	0	100
6	Group F	1.6	40–60	Individual and Cluster of rhombohedral	0	100
7	Group G	3.2	35–55	Individual and Cluster of rhombohedral	0	100

*NUMC* Native Ureolytic Microbial Community, *NA* not applicable.

**Table 4:** The morphology and phase characterization of CaCO_3_ crystals—bioaugmentation.

S. No	Group ID	Concentration of NUMC OD @ 600 nm	Concentration of S. *pasteurii* OD@ 600 nm	The average size of the crystal (µm)	Shape	Phase
1	Group 1	0	0.4	5–10	Rhombohedral	Calcite
2	Group 2	0.1		5–10	Rhombohedral	
3	Group 3	0.2		5–10	Rhombohedral	
4	Group 4	0.4		5–10	Rhombohedral	
5	Group 5	0.8		15–25	Individual and cluster of rhombohedral	
6	Group 6	1.6		30–40	Cluster of rhombohedral	
7	Group 7	3.2		30–40	Cluster of rhombohedral	

*NUMC* Native Ureolytic Microbial Community.

## Discussion

In this study, we investigated the effect of NUMC at varying concentrations on the biocementation potential of the most widely used biocementing culture *S. pasteurii*. We also compared the CaCO_3_ precipitation potential of NUMC at different concentrations in the presence and absence of *S*. *pasteurii*. The soluble calcium concentration was measured, and its kinetics was analysed using a logistic Eq. (4) to compare the biocementation potentials of NUMC and augmented *S*. *pasteurii*. pH was also monitored to identify the range that favours CaCO_3_ precipitation. SEM and XRD analyses were performed, which revealed the morphology (size and shape) and mineralogy of the crystals formed.

NUMC is capable of inducing CaCO_3_ precipitation in their microenvironment^38^. In Fig. 1a, the soluble calcium concentration decreased in all the groups. It could be due to carbonate ions generated in the MICP process during urea hydrolysis, which facilitates precipitation of soluble calcium around the bacterial cell wall in a cementation medium^2^. The complete exhaustion in the soluble calcium ions in the groups (group B–G) indicates that all the calcium in the medium is converted into CaCO_3_. Moreover, the supplied equimolar concentration of urea is enough for the complete conversion of CaCO_3_. The observed decrease in CaCO_3_ precipitation rate (Fig. 1a) is due to encapsulation of CaCO_3_ on the bacterial surface that limits the transport of nutrients transport including urea across the bacterial membrane^39^. The rate of soluble calcium depletion was observed to increase on increasing the NUMC concentration in the cementation medium. Increasing the NUMC concentration increases the total urease activity of the system, which in turn increases the soluble calcium depletion rate^22^. Moreover, the results show a positive correlation between CaCO_3_ precipitation rate and the cell concentration^22–24^. Furthermore, the relationship between K_cal_ and NUMC concentration could be used to design and develop a similar process for field applications. The kinetic constant K_cal, Max_ in the mathematical Eq. (3) denotes the maximum ability of the NUMC to achieve MICP at a faster rate, in this case, 0.1 h^−1^. The kinetic constant K_x_ is equal to 1 OD, which indicates the concentration of NUMC required to achieve half the value of K_cal, Max_.

*S*. *pasteurii* is a widely employed bacterial strain for bioaugmentation of soil consolidation and stabilization process because of its high urease-producing potential^40^. Hence, this bacterium was chosen as the model organism for this study. Supersaturation Index (SI) is one of the key parameters for the initiation of CaCO_3_ precipitation^5^. Quick CaCO_3_ precipitation was observed for groups 1–7 in the cementation medium. This indicates that the cementation medium has reached the required SI in a short time. pH also affects the SI, which is evident from the reported result^41^ (Fig. 2c). Moreover, the ready availability of the positively charged calcium ions in the vicinity of the negatively charged bacterial surface could also favour quick CaCO_3_ precipitation^3^.

The observed K_cal_ value of group 1 (0.64 h^−1^) with *S*. *pasteurii* of 0.4 OD was sixfold higher than the K_cal, Max_ (0.1 h^−1^) value of NUMC. This indicates that *S*. *pasteurii* has relatively high CaCO_3_ precipitation potential compared to NUMC. However, the observed results are in contrast to the reported studies that suggest biostimulation is the best possible approach for biocementation^38,42^. This could be due to the presence of different NUMC and varying study conditions between different research groups. The influence of varying concentrations of NUMC on the bioaugmentation potential of *S. pasteurii* was also investigated. However, no significant changes in the K_cal_ values were observed within the groups when K_cal_ values were compared between groups 1 to 7 (Fig. 2b). This indicates that the presence of NUMC did not influence the CaCO_3_ precipitation potential of *S. pasteurii* even at a concentration as high as eightfold (group 7) over a period of two weeks in this study.

The pH of the cementation medium greatly influences the CaCO_3_ precipitation and also affects bacterial urease production^41^. In this study, the pH of the cementation medium of all the groups irrespective of the group type varied between 6.5 to 8.3. This indicates that the CaCO_3_ precipitation occurred between the observed pH range. Urease activity of the bacteria results in the generation of ammonium ions that in turn affects the pH of the cementation medium. The rate of pH change was observed to be comparatively high for groups 1 to 7, which could be attributed to the high urease activity of *S. pasteurii*^43^. However, the same was not observed in groups A to G which could be attributed to the low urease activity of NUMC.

The molecular mechanism of CaCO_3_ crystal nucleation, growth, and morphology (size and shape) in the biocementation process is a complex phenomenon. Nature of the bacterial community, solution chemistry of the cementation medium (supersaturation index), the concentration of nutrients, calcium, and Mg^2+^ ions significantly influence the crystal growth kinetics and characteristics^44,45,47^. In this study, groups B to G with only NUMC at different concentrations showed a cluster of rhombohedral-shaped crystals, sized 35–100 µm at 288th hour. Whereas groups 1 to 4 with *S*. *pasteurii* in particular, yielded individual crystals of size 5–10 µm at 12th hour. A decrease in crystal size during bioaugmentation is due to the high driving force, which results in the fast attaining of the saturation state during CaCO_3_ precipitation. According to the classical nucleation theory: the nucleus size of the crystal decreases when the driving force to reach the saturation state for the precipitation increases^46^. This result is consistent with a previous study by Cuthbert and co-workers who reported that a higher initial saturation state influences the lower-sized crystals^39^.

The generation of ammonium ions and inorganic carbon due to the effective urea hydrolysis increases the pH and alkalinity of the cementation medium. It develops the oversaturated cementation solution that leads to the spontaneous CaCO_3_ precipitation^5^. It is possible to obtain different phases of CaCO_3_ including aragonite, calcite, vaterite, and two hydrated crystalline phases as monohydric calcite and ikaite in the MICP process^1^. This is because the polymorphism of CaCO_3_ is highly dependent on various parameters of the precipitation environment^47,49–51^. In general, many studies reported that the phase transition from metastable vaterite phase to more stable calcite phase during the CaCO_3_ precipitation process^22,26^. But, the specific phase preference by different bacterial cultures could depend on several parameters including the type of bacteria, specific amino acid sequences of urease, organic acid production, extracellular polymeric substances of the bacteria, the kinetics of the precipitation process, cementation medium composition, and other physicochemical parameters that affect supersaturation index of the solution^47–51^.

In this study, no visible CaCO_3_ crystals were observed in group A due to a lack of bacterial metabolic activity that leads to the undersaturation of the system. In the case of group B, besides 97.7% of calcite, 2.3% of vaterite form of CaCO_3_ crystals were formed at the end 288th hour. On the other hand, in all other groups including group C to G and group 1 to 7 only calcite form of CaCO_3_ crystals was observed at the end of precipitation (Fig. 4).

From the results, it is evident that calcite is the predominant polymorph of CaCO_3_ crystals in both cases. It is also evident that the presence of NUMC does not affect calcite formation. Moreover, the observed results follow the Ostwald rule of crystallization, which states that thermodynamically crystal formation favors the less soluble calcite than more soluble vaterite^27^. There could be a possible delay in the transformation of vaterite to calcite form when the rate of CaCO_3_ precipitation is slow. Hence, this could be attributed to the slow transformation of vaterite to calcite in groups B to G^27^. Nevertheless, only rhombohedral-shaped calcite form of crystals was observed in all the groups despite different bacteria employed in this study at the end of the process. These calcite form crystals have superior engineering properties (strength and stiffness) compared to vaterite and aragonite forms of CaCO_3_ crystals.

## Conclusions

In this study, we investigated the influence of NUMC on the biocementation potential of the most widely used bacterial culture *S. pasteurii.* We evaluated the biogenic CaCO_3_ precipitation kinetics of NUMC at varying concentrations in the presence and absence of *S*. *pasteurii* along with its impact on the morpho-mineralogical characteristics of the precipitated carbonates. The concentration of cells has a major impact on the CaCO_3_ precipitation kinetics as well as morpho-mineralogical properties of precipitated carbonate crystals as observed in the case of NUMC. The rate of CaCO_3_ precipitation in the case of NUMC is very slow compared to *S. pasteurii*; and this can have a major impact on its application*. S. pasteurii* is highly efficient in biocementation even in the presence of native ureolytic cultures at different concentrations. CaCO_3_ precipitation kinetics of *S. pasteurii* was not found to impact significantly in the presence of NUMC; even when their concentration is eight folds higher. Although the rate of CaCO_3_ is low in the case of NUMC, but it has a positive impact on the quality of crystals. The size of calcite crystals in the case of NUMC with low metabolic activity is much higher (6–10 times) compared to smaller crystals formed by *S. pasteurii*. This demonstrates that it is crucial to have fundamental knowledge on the biocementation potential of native communities and the need for alternatives such as supplementation of *S. pasteurii*. The observed results of the current study demonstrate, for the first time, that the quantitative and qualitative properties of biocement can be tailored utilising the information of CaCO_3_ precipitation kinetics with native as well as augmented cultures. The findings of this study can pave way for several new possibilities for ureolysis driven biocementation in the area of advanced functional living materials.

## Materials and methods

### Bacteria, growth medium, and OD measurement

**Figure 5:**
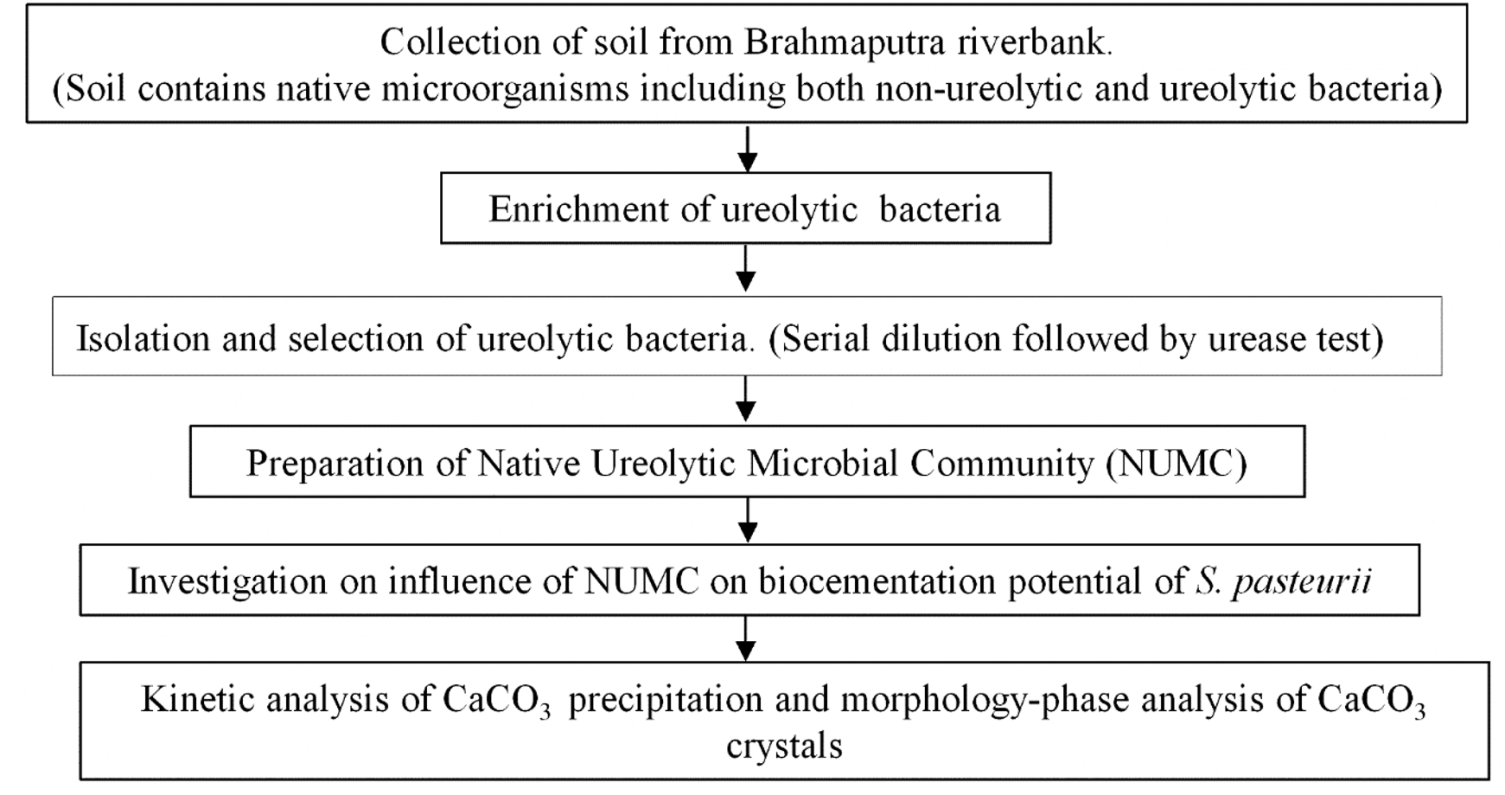
Schematic diagram of this study.

The bacteria used in this study are the Native Ureolytic Microbial Community (NUMC)^52^ and *S. pasteurii (ATCC 11859)*. The NUMC is a mixture of four different ureolytic bacteria (BS1, BS2, BS3, and BS4) in equal proportions isolated from Brahmaputra riverbank soil (India) and enriched using a medium containing 13 g/L nutrient broth and 5% urea. From the previous study^52^, the BLAST analysis of the 16S rRNA sequence results showed that BS1 and BS2 are close relatives of *Sporosarcina siberiensis*. Whereas BS3 and BS4 are close relatives of *Sporosarcina pasteurii*, and *Sporosarcina soli,* respectively. The selected bacteria turned the urea agar base from yellow to pink colour within 12 h in the qualitative urease test. The steps are included in Fig. 5. Both *S. pasteurii* and NUMC were grown in Ammonium -Yeast extract medium (ATCC 1376) contains yeast extract (20 g/L), ammonium sulphate (10 g/L), and 0.13 M tris base (pH 9) were maintained at 30 ºC and 180 rpm. The individual components of the growth medium were autoclaved and mixed after cooling under sterile conditions. To measure the concentration of the overnight grown NUMC and *S*. *pasteurii,* the media containing bacteria were centrifuged at 4500 rpm for 10 min and the optical density was measured using a spectrophotometer (Thermo scientific, Genesis 10S) at 600 nm with 0.85% sodium chloride solution as blank.

### Cementation medium and conditions

The cementation medium provides required nutrients and cementation components for NUMC and *S*. *pasteurii*. 100 mL of cementation medium was prepared by mixing 65 mL of autoclaved distilled water containing 0.2 g of yeast extract followed by the addition of required concentrations of NUMC and *S*. *pasteurii* cell pellet obtained after centrifugation (4500 rpm for 10 min). Then 10 and 25 mL of filter-sterilized 5 M urea and 2 M calcium chloride dihydrate solution were added, respectively. The cementation medium containing a bacterial pellet was maintained at 30 ºC and 180 rpm in a shaker incubator.

### Enumeration of bacterial concentration

The bacterial concentration was measured by the serial dilution method. Petri plates containing 1.5% agar in ATCC 1376 media were used to spread the bacteria; 1 OD of bacteria in saline was found to contain cells equivalent to 4.5 × 10^8^ cells/mL.

### Study design

This study was designed to investigate the influence of NUMC on the biocementation potential of augmented *S. pasteurii*. The study was divided into two major groups. Each group is further subdivided into seven subgroups namely A to G and 1 to 7. The groups A, B, C, D, E, F, and G have overnight grown NUMC pellet mixed with cementation medium at concentrations of 0, 0.1, 0.2, 0.4, 0.8, 1.6, and 3.2 OD, respectively. The groups 1, 2, 3, 4, 5, 6, and 7 contain fixed concentration of *S. pasteurii* (0.4 OD) and NUMC at concentrations of 0, 0.1, 0.2, 0.4, 0.8, 1.6, and 3.2 OD, respectively in the cementation medium. To monitor the process, 2 mL of samples were taken and centrifuged at 3000 rpm for 10 min at regular intervals of time. The obtained supernatant was used to measure soluble calcium concentration and pH until the process was complete.

### Measurement of soluble calcium ions and pH

The soluble calcium ions were measured by using the complexometric titration procedure^53^. 40 µL of the sample was diluted to 10 mL followed by the addition of 400 µL 1 N sodium hydroxide solution and a few drops of hydroxy naphthol blue disodium salt (1% W/V) solution indicators. Then the mixture was titrated against 1 mM EDTA disodium salt solution until the colour change from pink to blue was observed. The slope of the standard (0–2.5 mM CaCl_2_) was used to calculate the actual concentration of calcium ions in the sample. The change in pH during biocementation was recorded using a pH meter (Thermo scientific, Orion star, A211).

### Morphology and phase analysis of CaCO_3_

The CaCO_3_ precipitate from the cementation medium was analysed at the end of the process. 30 mL of sample was taken was centrifuged at 4500 rpm for 10 min. The pellets obtained were washed twice with distilled water and dried at 37 ºC overnight. Then the dried crystals were subjected to scanning electron microscopy and XRD.

### Morphology (size and shape)

The variable pressure electron microscope (VP-SEM, Zeiss, EVO 40-XVP, 2008) was used to observe the size and shape of the CaCO_3_ precipitate. The samples were placed on carbon-aluminum tape and coated using a carbon evaporative coater (Creissington, 2080C, 2011). The beam intensity and voltage were 8.0 and 10 kV, respectively with a working distance of around 15 mm. The secondary electron imaging was used to obtain scanning electron micrographs. The sizes of the crystals from the micrographs were obtained using IMAJEJ (1.8.0 172) software.

### Phase

Bruker D8 advance diffractometer with Ni-filtered Cu Kα radiation (40 kV, 40 mA) over the range 7°–120° 2θ, with a step size of 0.015° was used to collect the XRD data. The powdered CaCO_3_ was resuspended in ethanol and deposited onto low-background holders. Further, the phase identification was done in Bruker EVA 5.2 using the Crystallography Open Database (COD) (http://www.crystallogrphy.net/). The phase quantification was done in Topas Academic 7 using the Rietveld method. Also, the crystal structures were identified from the COD.

### Calculation of kinetic constants for calcium carbonate precipitation

Non-linear regression analysis was done using the curve fitting method. The exponential decrease of soluble calcium concentration over time was fitted with logistic Eq. (4) using the solver function in Excel (2016 MSO) to calculate the kinetic parameter called the kinetic constant of CaCO_3_ precipitation (K_cal_). In curve fitting, the least-square method was used. The sum of squared values of the difference between the experimental and predicted value was fixed as the objective function, and K_cal_ is the variable. K_cal_ values were further used to compare the kinetics of CaCO_3_ precipitation at various operating conditions of this study. The logistic Eq. (4) is a slight modification of Eq. (3) from our previous study^57^.

4 $${C}_{cal} \left(\mathrm{t}\right)=2{C}_{0}/ (1+{\mathrm{e}}^{{K}_{cal} t})$$

where, C_o_ is the initial concentration of calcium (mM), C_cal_ (t) is the soluble calcium concentration (mM) at given time, t is the time (h) and, K_cal_ is the kinetic constant of calcium carbonate precipitation (h^−1^).
